# Enhanced Bacteriocin Production by *Pediococcus pentosaceus* 147 in Co-culture With *Lactobacillus plantarum* LE27 on Cheese Whey Broth

**DOI:** 10.3389/fmicb.2018.02952

**Published:** 2018-12-03

**Authors:** Carolina Gutiérrez-Cortés, Héctor Suarez, Gustavo Buitrago, Luis Augusto Nero, Svetoslav Dimitrov Todorov

**Affiliations:** ^1^Instituto de Ciencia y Tecnología de Alimentos, Facultad de Ciencias Agrarias, Universidad Nacional de Colombia, Bogotá, Colombia; ^2^Instituto de Biotecnología, Facultad de Ciencias, Universidad Nacional de Colombia, Bogotá, Colombia; ^3^Departamento de Veterinária, Universidade Federal de Viçosa, Viçosa, Brazil

**Keywords:** bacteriocins, acid lactic bacteria, biopreservation, co-culture, cheese whey

## Abstract

The production of bacteriocins by lactic acid bacteria (LAB) has been of wide interest in the food industry due to their potential application in biopreservation. The production of bacteriocins is usually low in single strain fermentation, but can improve when the bacteriocinogenic strain is cultured in association with another bacteria. The present work aims to evaluate the growth and production of bacteriocins by *Pediococcus pentosaceus* 147 (bacteriocinogenic strain) in co-culture with *Lactobacillus plantarum* LE27 (inducer strain) using a culture medium based on cheese whey (CW). Strains were inoculated in co-culture in a CW broth at 7.24 Log CFU/mL of initial concentration of *P. pentosaceus* 147 and incubated at 37°C. Bacteriocin production was measured after 24 h by the critical dilution method, biomass was measured by plating on MRS agar (1% aniline blue), and a mono-culture was used as a control. The titers of bacteriocins produced by *P. pentosaceus* 147 in mono-culture were 19,200 AU/mL lower than those obtained in co-culture with *Lb. plantarum* LE27 at 51,200 AU/mL. The effect of adding the inducer strain at different times of incubation (3, 6, 9, and 12 h) was evaluated, with the addition of the induction factor at the beginning of the incubation of *P. pentosaceus* 147 generating the highest bacteriocin activity. This study shows the potential of inducing bacteriocinogenesis using co-cultures of strains of the genera *Pediococcus* and *Lactobacillus* and using alternative substrates such as cheese whey.

## Introduction

Bacteriocins are proteinaceous antimicrobial substances produced by Gram positive and Gram negative bacteria, with antagonistic activity against other, normally closely related, bacterial species (Taniguchi and Tanaka, 2004; Beshkova and Frengova, 2012). These substances are classified according to their molecular size, specificity of mode of action, presence of modified amino acids and morphological traits into four classes (Beshkova and Frengova, 2012; Zacharof and Lovitt, 2012). Bacteriocinogenic *Pediococcus* strains usually produce pediocin PA-1/AcH, a class IIa bacteriocin encoded by an operon composed of structural genes in addition to *pedB* (encodes the immunity peptide that protects the producer strain), *pedC* (codifies the ABC transporter), and *pedD* (codifies the complementary peptide to extracellular translocation) (Papagianni and Anastasiadou, 2009; Oppegard et al., 2015; Mesa-Pereira et al., 2017). The structure of this operon is similar to that described for plantaricins produced by *Lactobacillus plantarum* C11 (Diep et al., 1996) and *Lb. plantarum* NC8 (Maldonado et al., 2004), indicating that PA-1/AcH production can be modulated in the same way. Thus, pediocin production can be increased when the producer strain is co-cultured with other strains as reported by several authors (Maldonado et al., 2003; Bader et al., 2010; Man et al., 2012; Chanos and Mygind, 2016; Maldonado-Barragán et al., 2016).

Many investigations have been focused on the induction of bacteriocin production by the presence of another strain in the culture medium mediated by the quorum sensing system (QS), which is based on genetic expression synchronization (Chang et al., 2007; Ng and Bassler, 2009) to produce useful metabolites such as bacteriocins (Williams and Cámara, 2009; Pereira et al., 2013; Komnatnyy et al., 2014; Jia et al., 2017). The presence of additional bacteria in co-culture acts as a stress signal, and usually enhances the production of bacteriocin. Some studies have demonstrated that *Lactobacillus* spp. can act as an inductor improving the bacteriocin production of *Lactococcus lactis* and *Lb. plantarum* strains (Maldonado et al., 2004; Rojo-Bezares et al., 2007; Kos et al., 2011; Maldonado-Barragán et al., 2013). These studies have supported the concept of co-culture by which two or more strains are inoculated under aseptic conditions to obtain a metabolite of interest. These systems generate cell-to-cell interactions allowing stimulation of the production and expression of metabolites with industrial, medical and environmental applications (Goers et al., 2014). This has been previously shown in experiments with *Lactobacillus* strains; however, co-culture systems have not been evaluated on strains belonging to the genus *Pediococcus* sp. Based on the fact that plantaricin and pediocin are both encoded by similar operons, it will be possible to suggest that the co-culture system might be used to increase the bacteriocin production and expression by *Pediococcus pentosaceus* 147 [isolated form minas cheese and characterized as pediocin producer (Gutiérrez-Cortés et al., 2018)] as reported for *Lactobacillus* spp. cultures. This improvement in bacteriocin production might allow the development of an industrial production with applications on food preservation.

On the other hand, it is known that bacteriocin production by LAB (lactic acid bacteria) is highly affected by culture conditions as well as culture media composition (Todorov and Dicks, 2004; Sridevi et al., 2017), which must contain carbohydrates, organic nitrogen sources, amino acids, proteins, minerals and vitamins (De Arauz et al., 2012). Different studies have focused on the design of a new medium due to the high cost of the MRS broth in order to reduce the cost of bacteriocins production (Guerra et al., 2001; Liu et al., 2005; Kumar et al., 2012; Kaur et al., 2013; Suganthi and Mohanasrinivasan, 2015; Hati et al., 2017). In the last decade, research on bacteriocin production has focused on the development of simpler (and lower cost) culture media obtained from different natural sources such as molasses, corn syrup (Todorov and Dicks, 2006a,b), soy milk (Wolf-Hall et al., 2009), and cheese whey (CW) (Liu et al., 2005; Kumar et al., 2012; Prazeres et al., 2012; Hati et al., 2017) in order to obtain high amounts of bacteriocins with minimal cost and minimal usage of industrial waste products. CW is a highly nutritional matrix, containing more than half of the solids present in milk, with 6–10 g/L of proteins, 46–52 g/L of lactose, and minerals (Conti et al., 2012). Additionally, CW contains soluble vitamins and some enzymes that may be of interest to different industries (Prazeres et al., 2012), i.e., as raw material for biotechnological processes or for obtaining metabolites from bacterial growth (Sánchez et al., 2009). According to the literature, co-culture systems have been performed on commercial media MRS; therefore, the utilization of CW in the co-culture of LAB can be considered as a novel application of this kind of developed medium.

In the present study, a co-culture system was assessed in order to increase the bacteriocin production by *P. pentosaceus* 147 using *Lb. plantarum* LE27 as inductor strain. CW was also used as an alternative matrix for growth and bacteriocin production. The results confirm that induction by competition is applicable to *Pediococcus* strains and that CW is an optimal substrate for bacteriocin production in co-culture.

## Materials and Methods

### Bacterial Strains and Culture Media

*Pediococcus pentosaceus* 147 was previously isolated from artisanal Minas cheese produced with raw milk, and characterized as a bacteriocinogenic strain with inhibitory activity against different strains of *Listeria* spp. (Gutiérrez-Cortés et al., 2018) of 51,200 AU/mL when cultured in MRS broth (Oxoid, Basingstoke, England). The sugar fermentation profile of *P. pentosaceus* 147 was assessed by using API50CHL (bioMérieux, Marcy-l’Étoile, France) according to the manufacturer’s instructions. *Lb. plantarum* LE27 was previously isolated from molasses (Amortegui et al., 2014). *L. monocytogenes* 104 was previously isolated from poultry (Chiarini, 2007). LAB strains were stored in MRS broth and *L. monocytogenes* in brain, heart, and infusion (BHI, Oxoid), both supplemented with 20% glycerol (v/v) at −20°C. Before using aliquots of the stock, cultures were transferred to appropriate broths and incubated at 37°C for 24 h.

Cheese whey-based culture media were developed by using CW (CIMPA S.A.S., Bogotá, Colombia) and other compounds as described in Table 1. MRS broth (Oxoid) was considered as a control for bacterial growth and bacteriocin production.

**Table 1:** Composition of culture media developed for bacterial growth and bacteriocin production by *P. pentosaceus* 147 and *Lb. plantarum* LE27 (g/L).

Compound^1^	Modified MRS (g/L)	Cheese whey (CW) (g/L)
Peptone	10	19.8
Yeast extract	8	8
Meat extract	4	16
Whey^2^	20	30
K_2_HPO_4_	2	0.5
Tween 80	1	2
Ammonium citrate	2	2
Sodium acetate	5	1.5
MgSO_4_	0.2	0.2
MnSO_4_	0.04	0.04

*^1^All compounds, except whey, from Sigma-Aldrich (St. Louis, MI, United States); ^2^Cheese whey obtained from CIMPA S.A.S. (Bogotá, Colombia), with the following characteristics: humidity 43 g/Kg, ash 70.8 g/Kg, lactose 648.2 g/Kg, proteins 103 g/Kg, and fat 28.2 g/Kg; sodium 8183 mg/Kg, potassium 7484,5 mg/Kg, calcium 13116 mg/Kg, and magnesium 625.5 mg/Kg. Before use, pH of culture media was adjusted to 6.5 and autoclaved at 120°C for 15 min.*

### Antimicrobial Activity of *P. pentosaceus* 147

The overnight culture of *P. pentosaceus* 147 was diluted until an approximate concentration of 10^7^ CFU/mL was reached, and transferred individually to MRS (Oxoid) and modified MRS (Table 1), followed by incubation at 37°C for 24 h. Then, cultures were centrifuged at 8,000 × *g*, and the pH of the cell free supernatant (CFS) was adjusted to 6.5. The treated CFS was twofold diluted up to 1:256, and aliquots of 10 μL were spotted on a surface of a BHI medium with 1% agar (Oxoid), which was previously prepared and inoculated with *L. monocytogenes* 104 at 10^6^ CFU/mL. Inhibitory activity was expressed as arbitrary units/mL (AU/mL), considering the inverse of the highest dilution that presented an inhibitory zone higher than 2 mm in diameter (Gutiérrez-Cortés et al., 2018).

### Growth Dynamics and Bacteriocin Production on CW Broth on Mono-Culture

*Pediococcus pentosaceus* 147 and *Lb. plantarum* LE27 cultures were individually transferred to MRS and CW broth, at the end concentration of 10^6^ CFU/mL, and incubated at 37°C for 24 h. Every 3 h, aliquots of cultures were obtained, 10-fold diluted in NaCl 0.85 (w/v) and plated in duplicates on MRS agar, followed by incubation at 37°C for 24 h and with the results being expressed as CFU/mL. Aliquots were also treated as described previously to obtain CFS and tested for antimicrobial activity against *L. monocytogenes* 104, as described above. Tests were performed in three independent repetitions.

In addition, *P. pentosaceus* 147 was inoculated at final concentrations of 10^5^ and 10^6^ CFU/mL only on CW broth, and incubated at 37°C for 24 h. Bacterial growth and bacteriocin production were assessed every 3 h, as described above, in three independent repetitions.

### Growth Dynamics and Bacteriocin Production of *P. pentosaceus* 147 and *Lb. plantarum* LE27 Co-culture in CW Broth

Growth and inhibitory activity were measured in a co-culture of *P. pentosaceus* 147 and *Lb. plantarum* LE27 as described before. Strains were co-inoculated in CW at different concentration combinations (1: both at 10^6^ CFU/mL, and 2: *P. pentosaceus* 147 at 10^6^ CFU/mL and *Lb. plantarum* LE27 at 10^5^ CFU/mL) and incubated at 37°C for 24 h. Aliquots were obtained every 3 h, and CFS were prepared and tested for inhibitory activity against *L. monocytogenes* 104, as described above. Also, aliquots were 10-fold diluted in NaCl 0.85% (w/v), surface plated on MRS agar (Oxoid) supplemented with aniline blue (0.1%, w/v) (Brauman et al., 1996), and incubated at 37°C for 24 h. Colonies were enumerated considering their color (light blue for *P. pentosaceus* 147, and dark blue for *Lb. plantarum* LE27), and final results were expressed as CFU/mL. Tests were conducted in three independent repetitions.

### Adding of *Lb. plantarum* LE27 at Different Times as Pulses of Induction in the Bacteriocin Production by *P. pentosaceus* 147

Bacteriocin production of *P. pentosaceus* 147 was measured considering different co-inoculation periods and frequencies with *Lb. plantarum* LE27. Flasks containing 75 mL of CW were inoculated with 1.5 mL of *P. pentosaceus* 147 at an approximate final concentration of 10^6^ CFU/mL, being incubated at 37°C for 24 h. In different frequencies and times, aliquots of 1.5 mL of a *Lb. plantarum* LE27 culture (10^5^ CFU/mL) were added in the *P. pentosaceus* 147 CW systems (Table 2). After 24 h, the CFS of resulting cultures was obtained as described before, and the inhibitory activity against *L. monocytogenes* 104 was evaluated as described above. The experiment was conducted in three independent repetitions.

**Table 2:** Scheme of the inoculation (x) of *Lb. plantarum* LE27 and *P. pentosaceus* 147 in CW systems at different times and frequencies.

Treatment	*P. pentosaceus* 147	*Lb. plantarum* LE27			
	0 h	3 h	6 h	9 h	12 h
1 (A)	x	x	–	–	–
2 (B)	x	–	x	–	–
3 (C)	x	–	–	x	–
4 (D)	x	–	–	–	x
5 (E)	x	x	x	–	–
6 (F)	x	–	x	x	–
7 (G)	x	–	–	x	x
8 (H)	x	x	x	x	–
9 (I)	x	–	x	x	x
10 (J)	x	–	x	x	x
11 (control)	x	–	–	–	–

### Data Analysis

Antimicrobial activity (AU/mL) and microbial counts (log CFU/mL) were compared considering the tested conditions in the different assays, as described previously, by Analysis of Variance (ANOVA) and the Tukey test, using the software (R Core Team, 2015) and considering 5% to indicate significance.

## Results and Discussion

### Characterization of Carbohydrate Fermentation Profile of *P. pentosaceus* 147

The carbohydrate fermentation profile of *P. pentosaceus* 147 indicated an ability to metabolize lactose. This result confirmed that the strain was able to use lactose as an energy source, pointing to the adequacy of CW as an alternative culture medium for growth and bacteriocin production.

### Antimicrobial Activity of *P. pentosaceus* 147 on MRS and Modified MRS

**FIGURE 1:**
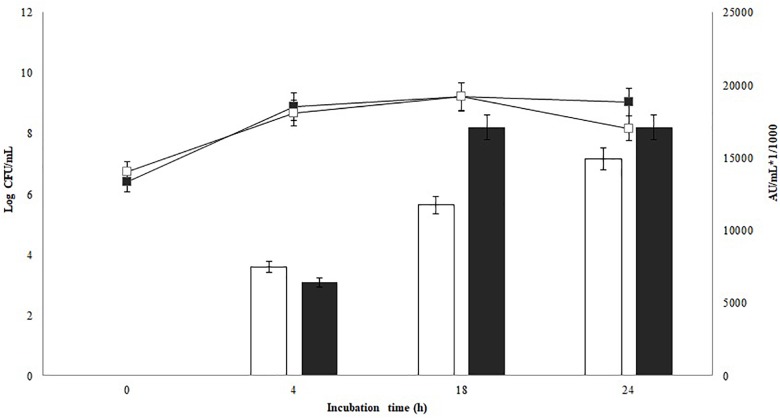
Growth and bacteriocin production kinetics of *P. pentosaceus* 147. Non-significant differences in the growth were found (*p* = 0.12). Bars represent antimicrobial activity (kAU/mL) (1 kAU/mL = 1000 AU/ml), non-significant differences in bacteriocin production (*p* = 0.33) were found; 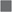: Modified MRS broth, 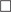: MRS broth.

The antimicrobial activity of *P. pentosaceus* 147 against *L. monocytogenes* 104 is shown in Figure 1. Non-significant differences in the growth of the bacteriocinogenic strain were found on tested culture media (*p* = 0.12). Comparing antimicrobial activity in MRS and modified MRS, a slight increase after 18 h of incubation was observed; nevertheless, non-significant differences were found (*p* = 0.33). Similar results have been reported for bacteriocin production by other *Pediococcus* spp. strains. Halami and Chandrashekar (2005) evaluated pediocin production of *P. acidilactici* C20 on whey broth supplemented with 2% of yeast extract and 0.1 of Tween^®^80, and compared this with MRS broth, a modified MRS and TGE (triptone-glucose-yeast extract) broth supplemented with 1 or 2% of lactose. *P. acidilactici* C20 showed a preference to metabolize 2% lactose as a carbon source on modified broth, and on whey supplemented with yeast extract as an additional source of nitrogen, the pediocin titer was 15,000 AU/mL, which was very similar to the results of the present work (Halami and Chandrashekar, 2005). On the contrary, *P. acidilactici* F presented a decreased production of bacteriocin on a modified MRS broth supplemented with lactose compared to MRS. Nevertheless, in milk, the activity of *P. acidilactici* F was intermediate, most probably related to the protein components of milk, which can be useful for bacteriocin synthesis (Somkuti and Steinberg, 2010). Turgis et al. (2016) reported more efficient pediocin production by *P. acidilactici* MM33 with glucose as a carbohydrate source (Turgis et al., 2016). Additionally, pediocin production increased when the bacteriocinogenic strain *P. pentosaceus* NCDC273 was inoculated in a modified MRS broth that contained glucose instead of lactose at 20 g/L (Vijay Simha et al., 2012). The results obtained in the present section showed that *P. pentosaceus* 147 is able to use lactose as carbon source and that the proteins and other components of the cheese whey can promote the production of bacteriocins, as evidenced in Figure 1.

### Growth Dynamics of *Lb. plantarum* LE27 and *P. pentosaceus* 147 Mono-Cultures in CW Broth

**FIGURE 2:**
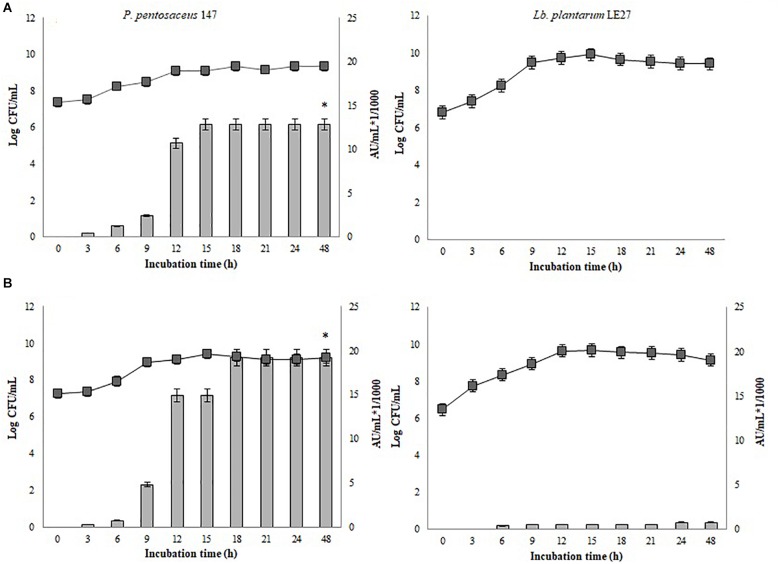
Growth and bacteriocin production kinetics of *P. pentosaceus* 147 (left) and *Lb. plantarum* LE27 (right). **(A)** MRS broth, **(B)** CW broth; 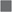: growth kinetic, non-significant differences in the growth of *P. pentosaceus* 147 (*p* = 0.08) and of *Lb. plantarum* LE27 (*p* = 1) were found; Bars represent antimicrobial activity (kAU/mL) (1 kAU/mL = 1000 AU/ml) of *P. pentosaceus* 147, significant differences (1.14e^-08^) were found (^∗^).

**FIGURE 3:**
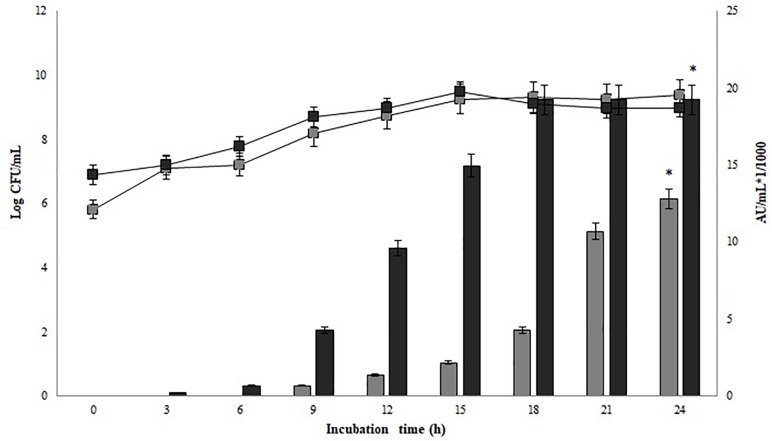
Growth and bacteriocin production kinetics *P. pentosaceus* 147 on CW broth. Inocula concentrations; 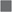: 10^5^ CFU/mL, 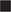: 10^6^ UFC/mL. Bars represent antimicrobial activity (kAU/mL) (1 kAU/mL = 1000 AU/ml), significant differences in bacteriocin production of *P. pentosaceus* 147 (*p* = 2.23e^-09^) were found (^∗^).

Figure 2 shows the kinetic growth and bacteriocin production of *P. pentosaceus* 147 and *Lb. plantarum* LE27. Non-significant differences in the growth of *Lb. plantarum* LE27 on MRS and CW broths were found (*p* = 1). *Lb. plantarum* LE27 exhibited antimicrobial activity at a concentration of 800 AU/mL against *L. monocytogenes* 104 when cultured in CW broth, while there was no antimicrobial activity in the MRS broth. That result suggests that CW can induce bacteriocin expression (Figure 2B, left). Non-significant differences in the growth of *P. pentosaceus* 147 in the MRS and CW broths were recorded (*p* = 0.8). Significant differences in antimicrobial activity (1.14e^−08^) in MRS and CW, and also using different biomass concentrations of *P. pentosaceus* 147 in the CW broth, were found (*p* = 2.23e^−09^). Figure 3 shows an antimicrobial activity of 19,200 AU/mL with an inoculum of 10^6^ CFU/mL ca. (final concentration) and of 12,800 AU/mL with 10^5^ CFU/mL ca. (final concentration) of inoculum.

Different studies have demonstrated that whey is an adequate substrate that supports the growth of *Lactobacillus* spp. strains as well as metabolite production, such as lactic acid (Panesar and Kennedy, 2011; Prazeres et al., 2012) or bacteriocins (Liu et al., 2005; Wolf-Hall et al., 2009; Garsa et al., 2014). Growth of *P. pentosaceus* 147 on the MRS and CW broths was very similar (Figure 2, right). Maximal antimicrobial activity on the MRS broth was 12,800 AU/mL while it was 19,200 AU/mL on the CW broth. Guerra et al. (2001) used concentrated and diluted whey in a culture of *P. acidilactici* NRRL B-5627, obtaining growth and bacteriocin production; the antimicrobial activity was higher than that obtained with *Lactobacillus* strains (Guerra et al., 2001), which was similar to the results of the present work. Although most *Pediococcus* spp. strains are not characterized for lactose fermentation (Papagianni and Anastasiadou, 2009), between 11% and 89% of *Pediococcus* strains are able to ferment lactose (Vos et al., 2009). Considering that the growth kinetics of *P. pentosaceus* 147 on the MRS and CW broths did not present significant differences (*p* = 0.8), it is possible to confirm that cheese whey is a good complex nutrient source for supporting the growth of LAB strains, as previously reported (Chaves de Lima et al., 2017).

### Growth Dynamics and Bacteriocin Production of *P. pentosaceus* 147 and *Lb. plantarum* LE27 Co-culture in CW Broth

**FIGURE 4:**
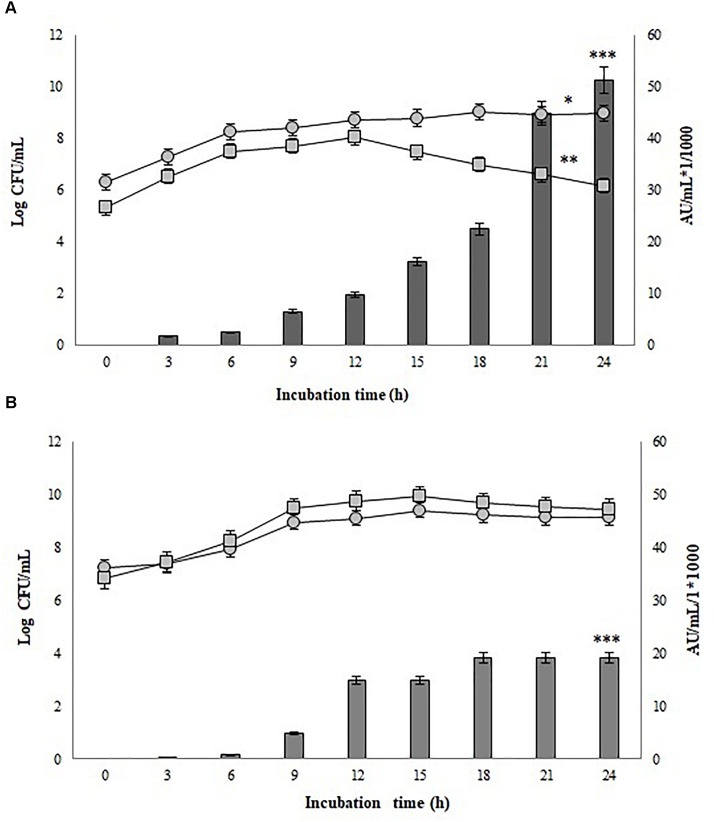
Growth and bacteriocin production kinetics of co-culture in CW broth; •: *P. pentosaceus* 147, 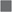: *Lb. plantarum* LE27. **(A)** *P. pentosaceus* 147 and *Lb. plantarum* LE27 co-culture, **(B)** Control in mono-culture in CW broth; •: Growth of *P. pentosaceus* 147, significant differences were found (*p* = 0.002) (^∗^). 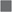: Growth of *Lb. plantarum* LE27, significant differences were found (*p* = 3.86^−5^) (^∗∗^). Bars represent antimicrobial activity (kAU/mL) (1 kAU/mL = 1000 AU/ml) of *P. pentosaceus* 147, significant differences (*p* = 0.03) were found (^∗∗∗^).

Significant differences in antimicrobial activity between the mono-culture of *P. pentosaceus* 147 and co-culture of *P. pentosaceus* 147 and *Lb. plantarum* LE27 were recorded (*p* = 0.003). As observed in Figure 4, the presence of *Lb. plantarum* LE27 (10^5^ CFU/mL) showed an increase from 19,200 AU/mL (in mono-culture) to 51,200 AU/mL (in co-culture) of bacteriocin production. Additionally, significant differences in antimicrobial activity, using 10^5^ and 10^6^ CFU/mL as an inoculum of the inductor strain *Lb. plantarum* LE27, were found (*p* = 0.02). On the other hand, in both cases, significant differences in growth kinetics were observed (*p* = 0.002) for *P. pentosaceus* 147 and (*p* = 3.86e^−5^) for *Lb. plantarum* LE27.

The strain of *Lb. plantarum* LE27 used in the present work as an inductor of bacteriocin production by *P. pentosaceus* 147 was reported as a bacteriocinogenic strain against some *L. monocytogenes* and *E. faecalis* strains (Amortegui et al., 2014). However, *Lb. plantarum* LE27 did not present any antimicrobial activity against *L. monocytogenes* 104 on the MRS broth and only very low antimicrobial activity on CW. According to the results presented in Figure 2 (mono-culture), the activity of *P. pentosaceus* 147 was 19,200 AU/mL, which shows a reduction from the initial titers obtained immediately after isolation (51,200 AU/mL) (Gutiérrez-Cortés et al., 2018).

As bacteriocin production by a producer strain is generally basal, if the cellular density is bigger in the lag and exponential phases of growth, the extracellular bacteriocin concentration can be higher (Brurberg et al., 1997; Tabasco et al., 2009). Similar results were reported with an inoculum of 10^7^ CFU/mL of *Lb. plantarum* LPCO10 in comparison to 10^5^ CFU/mL (Leal-Sánchez et al., 2002). Abbasiliasi et al. (2016) performed an optimization of an inoculum of *P. acidilactici* Kp10 between 1 and 10% and found maximal activity with 3% of inoculum. Nevertheless, the authors concluded that inoculum size had less importance than pH or incubation temperature on bacteriocin production (Abbasiliasi et al., 2016). Similar results were also observed for *Lb. acidophilus* LF221 and *Carnobacterium piscicola* A9b (Himelbloom et al., 2001). However, *Lc. lactis* subsp. *lactis* ST1 showed maximal activity with 10^5^ inoculum and less activity with higher concentrations (Taheri et al., 2012). In the present work, the inoculum that generated the maximal antimicrobial activity (10^6^ CFU/mL) in the co-culture was selected for further analysis.

In the mono-culture, *P. pentosaceus* 147 presented reduced antimicrobial activity and, in the case of *Lb. plantarum* LE27, was undetectable. That behavior, presented after the purification of strains from some microbial systems, could be explained by the absence of an inductor strain as a stimulus for the other strain to express some survival strategies, such as the production of antimicrobial substances in response to population density (Cornforth and Foster, 2013). Therefore, the antimicrobial activity of pure cultures decreases drastically in culture systems without competition. This behavior makes bacteriocin production an unstable trait of bacteria, being associated with the environment, and which tends to decrease and disappear under different laboratory conditions (Maldonado et al., 2004).

The aim of the present work was to increase bacteriocin production by the co-culture of *P. pentosaceus* 147, with *Lb. plantarum* LE27 as an inductor strain, since phylogenetic closeness generates the possibility that the sensor system of *P. pentosaceus* 147 could detect the extracellular signal peptides produced by *Lb. plantarum* LE27 on the CW broth, as previously suggested (Chanos and Mygind, 2016). This kind of culture generates interactions between the two populations by establishing cell–to-cell interactions, similar to the natural competitive conditions in the environment (Goers et al., 2014).

For the pure culture of *P. pentosaceus* 147 in the CW broth, antimicrobial activity over *L. monocytogenes* 104 of CFS was 19,200 AU/mL (Figure 3). In the case of the co-culture of *P. pentosaceus* 147 (10^6^ CFU/mL) and *Lb. plantarum* LE27 (10^5^ CFU/mL) on the CW broth, the antimicrobial activity titer was 51,200 AU/mL (Figure 4). As observed in Figure 4, the presence of *Lb. plantarum* LE27 (10^5^ CFU/mL) determined a difference of 19,200 AU/mL (in pure culture) to 51,200 AU/mL (in co-culture) of bacteriocin production by *P. pentosaceus* 147. These results can be linked to the QS mechanism, as reported in different studies (Maldonado et al., 2004; Chang et al., 2007; Straume et al., 2007; Man et al., 2012; Calasso et al., 2013; Maldonado-Barragán et al., 2013). The role of QS on bacteriocin production has been studied in more detail on *Lb. plantarum* strains; the bacteriocinogenic *Lb. plantarum* NC8 has been used in co-cultures with different genera of LAB as inducers due to the production of inductor peptides on the broth after incubation, and allowing the regulation of expression as a control of the synthesis of bacteriocin mediated by autoinductors (Maldonado et al., 2004).

Table 3 shows the specific growth rate and antimicrobial activity of both strains in mono and co-cultures. It was noticed that the final biomass presented differences in both cases. In the co-culture, the decrease in the *Lb. plantarum* LE 27 biomass was larger due the effect of the bacteriocins suspended in the medium. In Figure 4, growth kinetics show similar behaviors of *P. pentosaceus* 147 in mono- and co-culture. Nevertheless, significant differences were found (*p* = 0.02), therefore it is possible to conclude that in this case the co-culture had a negative effect on bacterial growth because of the small reduction that was observed. Mathys et al. (2009) reported no increase in the biomass of a co-cultured *Bifidobacterium thermophilum* RBL67 and *P. acidilactici* UVA1 (Mathys et al., 2009), and the same was also found for *Lb. plantarum* DC400 co-cultured with *L. plantarum* DPPMA20 and *Lb. sanfranciscensis* DPPMA174 (Calasso et al., 2013). Contrasting results have been reported with the *Lb. plantarum* KLDS1.0391 strain, where the biomass increased after co-culturing with different LAB strains due to the existence of a direct relation between biomass and antimicrobial activity (Man et al., 2012).

**Table 3:** Specific growth rate and antimicrobial activity of mono culture and co-culture.

	*P. pentosaceus* 147				*Lb. plantarum* 147			
	Mono-culture		Co-culture		Mono-culture		Co-culture	
	
Time (h)	Log CFU	AU/mL	Log CFU	AU/mL	Log CFU^∗^	AU/mL	Log CFU^∗^	AU/mL
	7.24 ± 0.32	0 ± 0.0	6.27 ± 0.33	0 ± 0.0	6.46 ± 0.06	0 ± 0.0	5.3 ± 0.43	–
	7.36 ± 0.09	266 ± 0.14	7.27 ± 0.10	1600 ± 0.0	7.74 ± 0.089	0 ± 0.0	6.5 ± 0.86	–
	7.92 ± 0.21	733 ± 0.05	8.26 ± 0.21	2400 ± 0.21	8.35 ± 0.25	400 ± 0.0	7.49 ± 0.58	–
	8.94 ± 0.11	4800 ± 0.00	8.42 ± 0.12	6400 ± 0.0	8.94 ± 0.17	533 ± 0.14	7.69 ± 0.12	–
	9.10 ± 0.01	14933 ± 0.08	8.69 ± 0.01	9600 ± 0.0	9.64 ± 0.06	533 ± 0.14	8.01 ± 0.67	–
	9.40 ± 0.03	14933 ± 0.08	8.78 ± 0.03	16000 ± 0.12	9.67 ± 0.04	533 ± 0.14	7.46 ± 0.18	–
	9.24 ± 0.11	19200 ± 0.0	9.03 ± 0.11	22400 ± 0.08	9.56 ± 0.11	533 ± 0.14	6.98 ± 0.98	–
	9.12 ± 0.35	19200 ± 0.0	8.93 ± 0.04	44800 ± 0.08	9.51 ± 0.02	800 ± 0.0	6.58 ± 0.47	–
	9.13 ± 0.01^∗^	19200 ± 0.0^∗∗^	8.96 ± 0.14^∗^	51200 ± 0.0^∗∗^	9.42 ± 0.05^∗^	800 ± 0.0	6.14 ± 0.14^∗^	–

*^∗^Significant differences in kinetics growth were found (p < 0.05). ^∗∗^Significant differences in bacteriocin production by P. pentosaceus 147 were found (p < 0.05).*

Rojo-Bezares et al. (2007) reported that no antimicrobial activity from *Lb. plantarum* J23 was observed in a pure culture. Even so, after 6 h of co-culture with *Lc. lactis* MG1363 and *Lb. hilgardii* J8, the strain showed activity; the same authors also reported that contact with living inducer cells was necessary for the development of activity, and CFS or dead cells did not induce antimicrobial activity in *Lb. plantarum* J23 (Rojo-Bezares et al., 2007). The non-induction by the addition of CFS or dead cells has been explained by the fact that inductor molecules could not be released into the culture medium and, therefore, remained in the intracellular space or associated with the membrane, and could not be sensed by bacteriocinogenic cells (Chanos and Mygind, 2016). However, it has been demonstrated that in some cases, such as in the production of plantaricin PlnA by *Lb. plantarum*, cell-to-cell contact was not necessary to increase antimicrobial activity (Di Cagno et al., 2010). Different bacterial species are able to increase the bacteriocin production. Plantaricin production by *Lb. plantarum* KLDS1.0391 increased in the presence of different strains from genera *Lactobacillus*, *Lactococcus*, *Leuconostoc*, *Streptococcus*, and specially *Enterococcus* (Man et al., 2012). Another study used different strains of *Lb. plantarum* in co-culture with LAB strains and found that *Lc. lactis* ssp. *lactis* IL1403 was the only one that was able to act as an inductor with all *Lactobacillus* spp. strains, suggesting that the ability of induction depends on the particular subspecies (Maldonado-Barragán et al., 2013).

**FIGURE 5:**
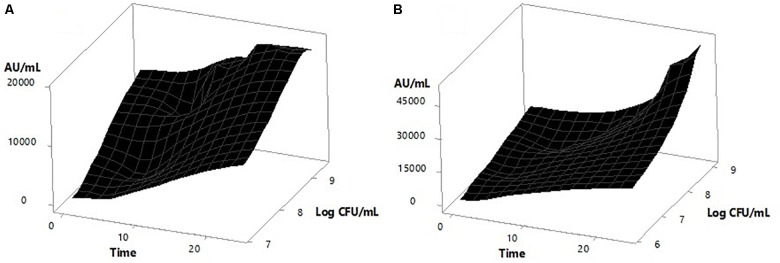
Surface response graphs corresponding to *P. pentosaceus* 147 growths and antimicrobial activity during the incubation time. **(A)** Mono-culture and **(B)** Co-culture.

Figure 4 also shows that the inducer strain apparently had some sensitivity to high concentrations of the peptide, since a decrease in biomass was observed after 15 h of incubation when the bacteriocin titer was 16,000 AU/mL with 10^5^ CFU/mL of inoculum, and after 18 h was 19,200 AU/mL with 10^6^ CFU/mL. However, the biomass reduction (which was significant *p* = 3.86e^−5^) was not high enough to stop metabolite production, obtaining a final activity of 51,200 AU/mL in the first case and 38,400 AU/mL in the second case. Figure 5 shows the surface response graph that represents the antimicrobial activity and biomass behavior during incubation. These results differ from those obtained by *Lb. plantarum* J23 in co-culture with *Lb. hilgardii* J81 which, being susceptible, disappeared in the medium, generating a decrease in bacteriocin production at the end of incubation (Rojo-Bezares et al., 2007). The pediocin produced by *P. pentosaceus* 147 corresponds to the fragment of 1,044 bp (Gutiérrez-Cortés et al., 2018), reported as pediocin PA-1/AcH (Marugg et al., 1992); according to this theory, its expression is regulated by QS. As a consequence, the present work has demonstrated that it is possible to obtain higher expression of the bacteriocin by *P. pentosaceus* 147 by the presence of an inducer, in this case *Lb. plantarum* J23.

### Addition of *Lb. plantarum* LE27 at Different Times as Pulses of Induction in the Bacteriocin Production by *P. pentosaceus* 147

**FIGURE 6:**
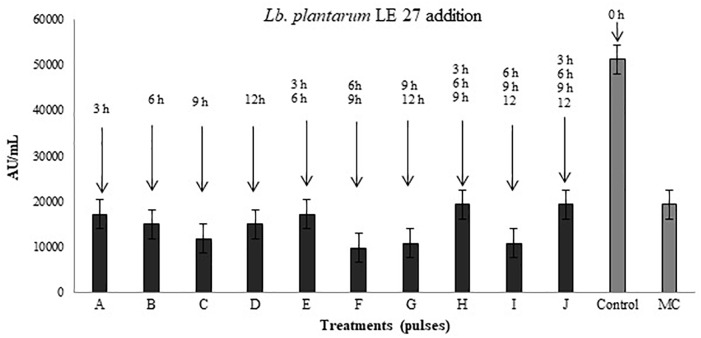
Antimicrobial activity after addition of induction pulses of *Lb. plantarum* LE27 to *P. pentosaceus* 147 culture. A: 3 h; B: 6 h; C: 9 h; D: 12 h; E: 3 and 6 h; F: 6 and 9 h; G: 9 and 12 h; H: 3, 6, and 9 h; I: 6, 9, and 12 h; J: 3, 6, 9, and 12 h. Control: co-culture of *P. pentosaceus* 147 and *Lb. plantarum* LE27 inoculated at the same time (0 h). MC: *P. pentosaceus* 147 in mono-culture.

Figure 6 shows the antimicrobial activity of *P. pentosaceus* 147 co-inoculated with *Lb. plantarum* LE27 at different times and frequencies. The treatment that produced the highest titer of bacteriocins of 51,200 AU/mL was the control, which means that the optimal moment to add the inducer strain is at the same time the inoculation of the producer strain starts the co-culture incubation.

Figure 6 also shows that in treatments where the induction begins after 6 h of incubation (treatments C, F, G, I), *P. pentosaceus* 147 presented the lowest antimicrobial activity compared to the bacteriocin production in the pure culture. Similar results have been reported with the bacteriocinogenic strains *Lb. helveticus* M92, *Lb. plantarum* L4 and *E. faecium* L3, which were inoculated with the inducer strain *Lc. lactis* subsp. *lactis* LMG 9450, at the same time obtaining an increment in antimicrobial activity (Kos et al., 2011). The same occurred when *Lb. plantarum* NC8 was cultured at the same time with *Lc. lactis* MG1363 (Maldonado et al., 2004), and when *Lb. salivarius*, *E. durans*, *E. faecium*, *E. hirae*, and *E. faecium* were cultured at the same time with *Lb. crispatus* NRRL and *Lb. acidophilus* as inducers (Svetoch et al., 2010).

## Conclusion

The co-culturing of LAB has proven to be an alternative for the induction of bacteriocin production. In the case of *P. pentosaceus* 147, it has been shown that the presence of *Lb. plantarum* LE27 generates an increase in the production of pediocin, as evidenced in the antimicrobial activity against *L. monocytogenes* 104. This allows for the application of this type of induction as an alternative for research into the large-scale production of bacteriocin for industrial purposes. Additionally, in this study, it was shown that cheese whey can be considered as a promoter substrate for the expression of bacteriocins since an increase was observed with respect to activity in the MRS broth. In the present work, different induction times were evaluated, finding greater effectiveness when the inoculation of the two strains was performed at the same time. These results are at an initial phase of the research into the induction mechanisms, and must be followed by an understanding at the proteomic level of what occurs with the expression of genes related to the stimulation of pediocin synthesis by *P. pentosaceus* 147 in co-culture.

## Author Contributions

CG-C, HS, and GB conceived the idea. HS and GB directed the project. CG-C and ST carried out the experiments and drafted the manuscript. LN verified the experimental methods. HS, GB, and LN revised and approved the final manuscript.

## Conflict of Interest Statement

The authors declare that the research was conducted in the absence of any commercial or financial relationships that could be construed as a potential conflict of interest.
